# A rare presentation of ectopic thyroid tissue in the submandibular region: a case report

**DOI:** 10.11604/pamj.2021.39.217.27390

**Published:** 2021-07-28

**Authors:** Theresa Obermueller, Maximilian von Bernstorff, Bruno Valentin Sinn, Rakan Saadoun, Bastian Gebhardt, Veit Maria Hofmann

**Affiliations:** 1 Department of Otorhinolaryngology, Head and Neck Surgery, Charité-Universitätsmedizin Berlin, Corporate Member of Freie Universität Berlin, Humboldt-Universität zu Berlin and Berlin Institute of Health, Campus Benjamin Franklin, Berlin, Germany,; 2 Institute of Pathology, Charité-Universitätsmedizin Berlin, Corporate Member of Freie Universität Berlin, Humboldt-Universität zu Berlin and Berlin Institute of Health, Berlin, Germany,; 3 Department of Otorhinolaryngology, Head and Neck Surgery, Ruprecht-Karls-University Heidelberg, Faculty of Medicine Mannheim, Mannheim, Germany

**Keywords:** Ectopic thyroid tissue, submandibular mass, thyroid goiter, case report

## Abstract

Ectopic thyroid tissue in the lateral neck is a rare finding, especially in the submandibular region. This case report presents a 38-year-old female patient with swelling in the lateral cervical neck. Due to a thyroid goitre, right hemithyroidectomy was performed in the past. However, a persistent high thyroglobulin level was detected after surgery. Regarding the suspected tumour in the submental region, a cervical magnetic resonance imaging (MRI) was performed, which revealed a suspicious looking mass. The patient underwent complete surgical excision and the histopathological report concluded that the tumour was ectopic thyroid tissue. Her thyroglobulin level decreased back to a normal level after excision of the submandibular mass. These results show that ectopic thyroid tissue must be considered a differential diagnosis for patients with unclear swelling in the submental region.

## Introduction

The incidence of ectopic thyroid tissue is low, with approximately one case per 100,000 - 300,000 persons [1]. During embryogenesis, between weeks five and seven, thyroid gland tissue migrates from the base of the tongue (foramen cecum) down to the pretracheal neck region. Abnormalities in migration along the thyroglossal duct might result in ectopic thyroid tissue, usually in the midline between the tongue and the diaphragm [2]. This case report presents a patient with a rare finding of ectopic thyroid tissue in the submandibular region. Unlike ectopic tissue of the tongue base, this location is seldom seen [3].

## Patient and observation

**Patient Information:** a 38-year-old female patient presented with left submandibular swelling. The patient had detected the mass two years prior. She reported recurrent fever but denied weight loss or night sweats. In 2003, two nodes (one inhomogeneous, one hypoechoic) were detected in the left thyroid tissue. A scintigraphy, which did not include the submandibular region, revealed a cold thyroid node and normally functioning thyroid tissue in the common location. After scintigraphy and due to a thyroglobulin value greater than 350 ng/ml (normal <70 ng/ml), a follicular or papillary thyroid carcinoma was suspected. Therefore, a hemithyroidectomy was performed. Per the histopathological findings, a diffuse goitre was diagnosed. After the surgery, hypothyroidism was diagnosed. Therefore, L-Thyroxin 125 μg was prescribed to the patient. Post-surgery, the thyroglobulin level was persistently unexplainably high at 641 ng/ml. The patient was referred for further evaluation with an endocrinologist and a specialist in nuclear medicine.

**Clinical findings:** her clinical examination revealed a non-fixed and firm mass (3.00 * 2.00 cm) located in the left cervical level Ia. Endoscopic examination of the oropharynx, including the mouth and the tongue base and the larynx were unsuspicious.

**Figure 1:**
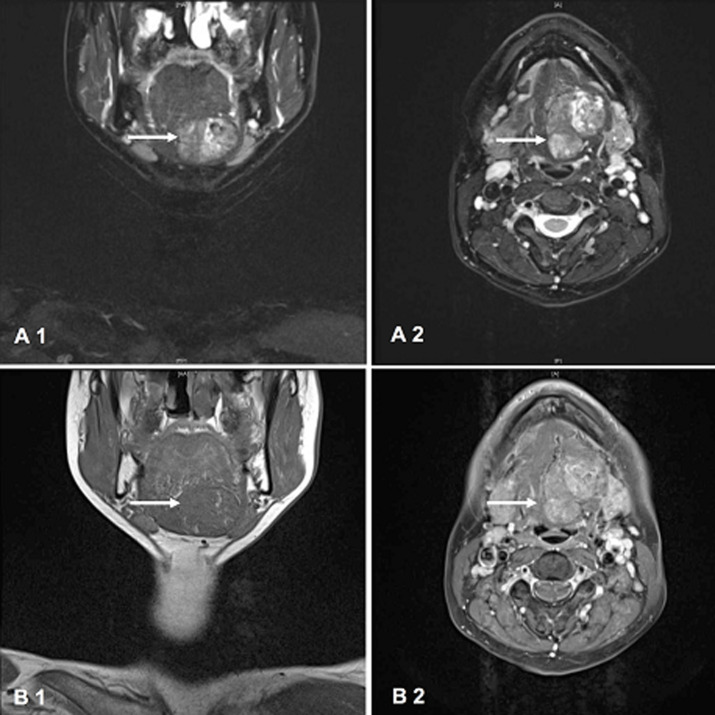
MRI scan showing a submental mass (white arrow): A1) T2-weighted (coronary); A2) T2-weighted (axial); B1) T1-weighted (coronary); B2) T1-weighted (axial)

**Diagnostic assessment:** ultrasound examination showed an inhomogeneous hypo- and hyperechoic mass (3.07 * 2.12 * 2.23 cm) with an anechoic surrounding. Furthermore, lymph nodes in levels II-IV on both sides were increased in size with normal morphology. The left thyroid tissue was missing after hemithyroidectomy and the right thyroid tissue was of non-suspicious morphology. The thyroid-stimulating hormone value was 0.55 mU/l (normal 0.27 - 4.20 mU/l) and the thyroglobulin level pre-surgery was 348.50 ng/ml (normal 3.20 - 59.70 ng/ml). An MRI scan of the neck showed an ill-defined mass of 3.00 * 4.00 * 2.50 cm located submentally left with an inhomogeneous T2W signal, suggesting malignancy (Figure 1). Initial differential diagnoses based on the patient´s history and clinical findings included papillary or follicular thyroid carcinoma, ectopic thyroid tissue, benign or malignant tumour of the left submandibular gland and a median cervical cyst.

**Figure 2:**
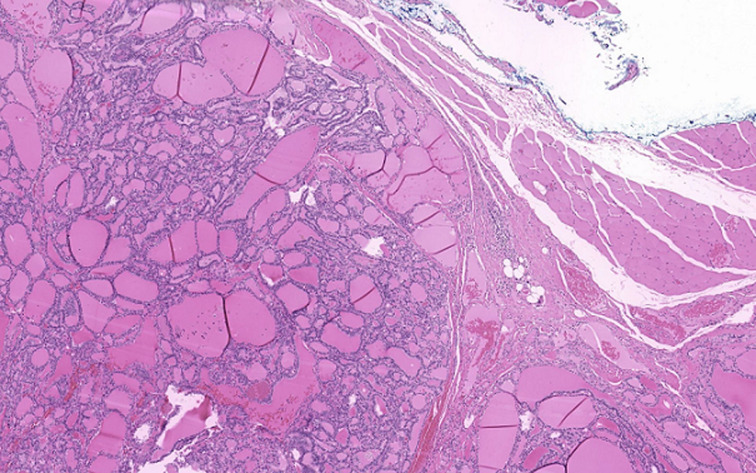
representative microscopic images of H&E stained biopsy of ectopic thyroid tissue

**Therapeutic intervention:** a surgical excision of the mass and the neighbouring hyoid bone was performed via a cutaneous approach under general anaesthesia. The left submandibular gland as well as the facial nerve were protected. The histopathological report revealed ectopic thyroid tissue without any malignancy (Figure 2).

**Follow-up and outcome:** one week after surgery, the patient´s thyroglobulin level was 55.1 ng/ml. Further adjustments in hormone therapy were performed by an endocrinologist (137 μg per day).

## Discussion

In the majority of patients, around 90% of ectopic thyroid tissue is located in the base of the tongue [4]. Submandibular thyroid tissue is extremely rare [3,5], and reports of it being located anywhere other than the midline are few in the literature [1]. Notably, ectopic thyroid tissue is more common among females [5]. Thyroglobulin is overexpressed in patients with autoimmune thyroid disease with benign and malignant thyroid tumours [6]. While malignancy in ectopic thyroid tissue is rare [7], it was suspected in our patient due to a persistent high thyroglobulin level.

On MRI scans, ectopic thyroid tissue is usually iso- to lightly hyperintensive in T1-weighted images and lightly hyperintensive in T2-weighted images [8]. In our patient, the mass was extremely hyperintensive and therefore a suspected malignancy.

In this type of case, a technetium-9 m or iodine-131 scintigraphy should be performed to identify possible ectopic thyroid tissue and functioning thyroid glands [9]. Analysing the function of the thyroid tissue is relevant for not inducing hypothyroidism after excision of the ectopic thyroid tissue. In nearly 70% of patients, only ectopic thyroid tissue is functioning [10]. The majority of previously reported cases of submandibular ectopic thyroid tissue showed only ectopic thyroid tissue and no normal thyroid tissue [3]. However, in our patient, functioning normal thyroid tissue was identified in a scintigraphy that was performed several years before the excision of the cervical mass. Furthermore, regarding the thyroid hormones our patient had already substituted L-thyroxin beginning with the first surgery.

## Conclusion

The presented case highlights that ectopic thyroid tissue should be considered a differential diagnosis for patients with unclear swelling in the lateral submandibular region. Furthermore, the relevance of further scintigraphy, including of the submandibular region, in cases of persistent high thyroglobulin is outlined.

**Informed consent:** informed consent was given by the patient.
